# Analysis of physical characteristics and mechanism of retarder to stratified cemented backfill

**DOI:** 10.1038/s41598-024-64507-5

**Published:** 2024-06-14

**Authors:** Chunming Ai, Anju Yu, Chao Liu, Tao Li

**Affiliations:** 1 College of Safety Science and Engineering, Liaoning Technical University, Huludao, 125000 China; 2 Key Laboratory of Thermal Disaster and Prevention, Ministry of Education, Huludao, 125000 China; 3 Norin Mining Limited, 12A Guang An Men Nan Jie, Beijing, 100053 China

**Keywords:** Stratified cemented backfill, Retarder, Physical characteristics, Mechanism, Energy science and technology, Engineering

## Abstract

The stratified structural plane caused by stratified backfill will reduce the strength of backfill, and the introduction of retarder will make up for the defect. Three retarders, sodium tripoly-phosphate, citric acid and sucrose, were introduced. After determining the optimal dosage of retarder, they were added into the filling slurry with a ratio of lime to sand of 1:6 and a mass concentration of 75%. Based on the hydration reaction mechanism and damage mechanics theory of cement, the setting time test and uniaxial compressive strength test were carried out. With the help of scanning electron microscopy and X-ray diffraction, the influence mechanism of retarder on the physical characteristics of stratified cemented filling was investigated.The main research contents and achievements are as follows:. The results showed that the three retarding agents can delay the setting time of the cement filling slurry, and the retarding effect is sucrose > citric acid > sodium tripolyphosphate. The addition of retarder can improve the uniaxial compressive strength and integrity of stratified consolidated backfill, and the best filling interval time (FIT) is 12 h. Appropriate addition of retarder will increase the amount of cement hydration products, make the structure of hydration products more dense, reduce the formation of stratified structural plane, and help to improve the strength of stratified cemented backfill.

## Introduction

**Figure 1:**
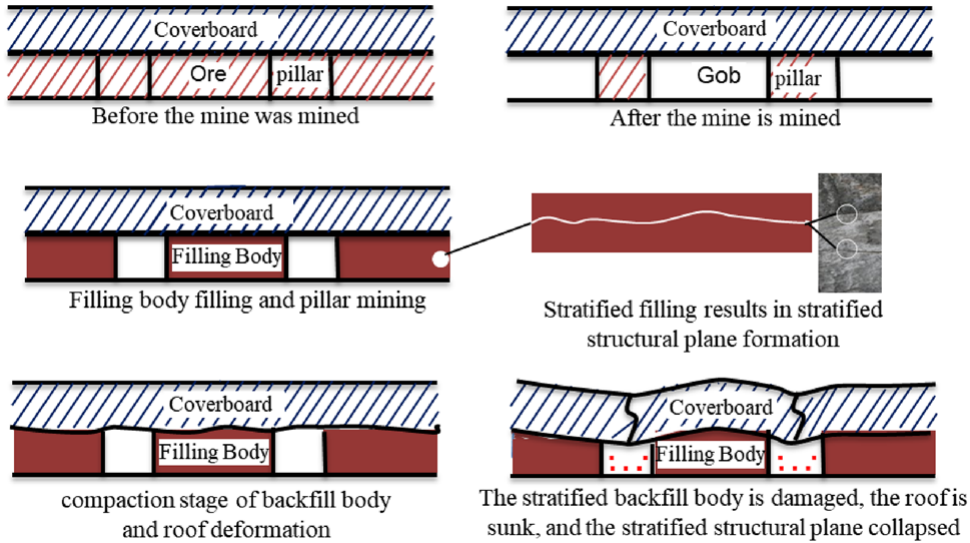
Cementing backfill mining process and deformation and failure process of cementing backfill body.

Filling method can not only solve the problems of tailing pollution and land occupation, but also improve the ground stability and increase the ore recovery rate. This method has social, economic and environmental benefits^1,2^. Due to production planning arrangements such as single filling quantity limitation, material preparation and transportation, regular maintenance of machinery and equipment, and strength requirements of filling retaining wall, stopes generally require several filling operations to complete filling, resulting in the appearance of stratified structural planes^3–5^. Filling at different intervals will reduce the strength of the backfill and the stability of the gob. Stratified structural plane will reduce the strength of cemented backfill^6–8^, easily lead to roof collapse and stratified structural plane caving, resulting in serious casualties and property losses^9^, As shown in Fig. 1. Tang, Y.N. et al.^10^ used uniaxial compression test and two-dimensional particle flow software (PFC-2D) to deeply analyze the influence of structural characteristics on the physical characteristics and crack evolution law of cemented filling. Shen, H.M.^11^ analyzed the failure mode of the graded cemented backfill and studied the law of the influence of placement times on the strength reduction of the cemented backfill. Kasap T et al.^12^ found the growth in solid concentration, curing time (up to 90 days) and the amount of sand in the mixtures (up to 30%) had a boosting consequence on the fill's performance. Guner N U et al.^13^ found calcareous sand made a major contribution to the filling strength, incorporating the effects of enhancing the fill gradation’s readjustment and reducing the sum of cement being used in the unit volume for CPB manufacturing. Cao, S. et al.^14,15^ studied the change rule between physical characteristics of cemented filling and filling times. Factors such as the number of layers of cemented backfill and filling Angle to explore the physical characteristics of cemented backfill and their relationship. Xu et al.^16^ studied the physical properties of stratified cemented backfill with different ratios, and evaluated the strength efficiency of the structural plane of cemented backfill.

Retarding agent is a kind of engineering additive that can reduce the hydration of cement. It can extend the setting time of cement-containing backfill, cement, gypsum and other materials from several hours to several days without affecting its durability^17,18^. Retarding agent can be used for ultra-long distance pipeline transportation, long-term transportation and long-term construction^19^. Yu, X. et al.^20^ studied the influence of hydroxyl and carboxyl groups in the molecular structure of hydroxy-carboxylic acid retarding agents on the hydration of cement. Guo, P. F. et al.^21^ tested the effects of five retarding agents, such as citric acid and sodium gluconate, on the setting time, compressive strength and flexure strength of cement paste, and analyzed their effects on cement hydration. Wu, J. G. et al.^22^ studied the influence of sucrose content on the setting time of Portland cement and the strength of cement paste when it changed in a wide range. The coagulation regulation mechanism of sucrose on Portland cement was discussed. Zhang, X. P. et al.^23^ studied the effects of different retarders on the setting time of cement and the strength of mortar. Jansen et al.^24^ found that the complexation of PCE with calcium ions was the main reason for the slow coagulation of PCE. The addition of PCE will complex calcium ions in the liquid phase, delay the formation of ettringite, and then lead to the slow dissolution of sulfate mineral phase and C_3_A.

The above researchers have made a lot of studies, but they have not proposed the solution measures to reduce the strength of backfill caused by stratified backfill, and have not studied the influence of retarding agents on the physical characteristics of stratified backfill. Therefore, this paper considers adding retarder to the filling slurry, and studies the effects of different retarder and retarder content on the setting time of backfill, the strength and failure form of backfill, so as to solve the formation of stratified structural plane caused by backfill and improve the strength of stratified consolidated backfill. On this basis, the enhancement effect of retarder is analyzed.

## Experimental materials

### Physical and chemical properties of tailings

**Figure 2:**
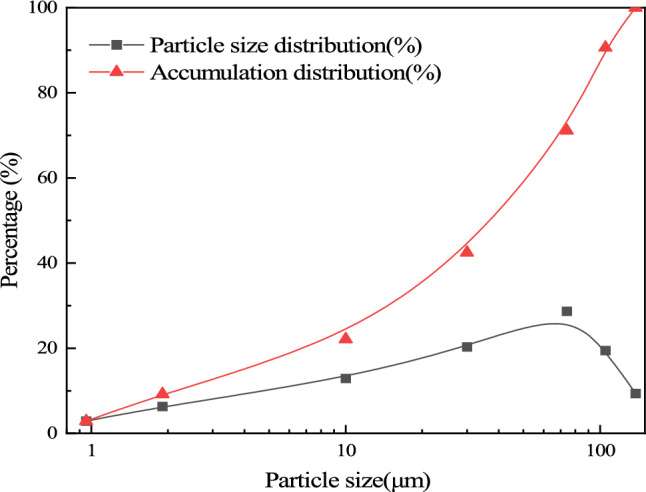
Grading curve of tailings.

The tailings discharged from the concentration plant of Bianjiadayuan lead–zinc-silver mine were used as filling materials, which were sampled on site and transported to the laboratory for storage. The pH value of the mine tailings is 9, the density is 2839 kg/m3, and the porosity is 47%. The grain size grading curve of tailings is shown in Fig. 2. The non-uniformity coefficient and curvature coefficient of tailings are 26.57 and 1.801. The particle size distribution of the tailings is wide and the grading is good. The chemical composition of tailings is shown in Table 1. The main components of the tailings are SiO_2_, Al_2_O_3_, CaO, MgO and other oxides, which can accelerate the formation of the strength of the backfill. The sulfur content is 3.44%, and its content is less than 10%, which will not weaken the strength of the backfill in the later stage, so the tailings can be used to prepare full tailings filling slurry.

**Table 1:** Chemical composition of tailings.

Element	Dosage/%	Element	Dosage/%	Element	Dosage/%
SiO_2_	56.20	Fe_2_O_3_	9.21	As_2_O_3_	0.13
Al_2_O_3_	16.14	ZnO	0.27	Rb_2_O	0.036
CaO	5.81	Na_2_O	0.44	SrO	0.019
MgO	2.58	P_2_O_5_	0.22	ZrO_2_	0.011
TiO_2_	0.74	Cr_2_O_3_	0.045	PbO	0.081
MnO	0.68	CuO	0.0097	Nb_2_O_5_	0.0013
K_2_O	3.76	Ir_2_O_3_	0.0051	Cl	0.0147
SO_3_	3.44	BaO	0.049	/	/

### Cement and retarding agent

The P.O42.5 Portland cement is selected according to the actual situation on site.The three commonly used retardants are hydroxy-carboxylic acid retarder citric acid (C_6_H_8_O_7_·H_2_O), sugar retarder sucrose (C_12_H_22_O_11_), and phosphate retarder sodium tripolyphosphate (Na_5_P_3_O_10_).

## Experimental method

### Setting time test of cemented backfill

**Figure 3:**
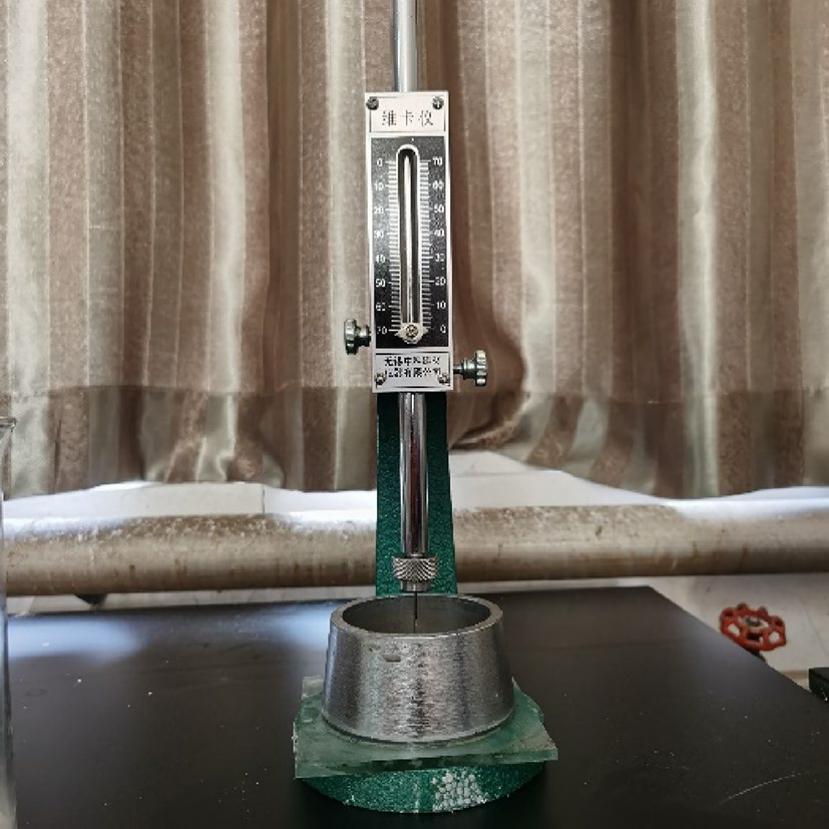
Vicat instrument.

The setting time of cemented filling paste mixed with different retarder contents was tested according to GB/T 1346–2011 "Test Method for Water Consumption, Setting Time and Stability of Cement Standard Consistency", and the test equipment Vicat instrument was shown in Fig. 3. The effect of different retarders on the setting time of filling slurry was evaluated.

### Strength test of cemented backfill

**Figure 4:**
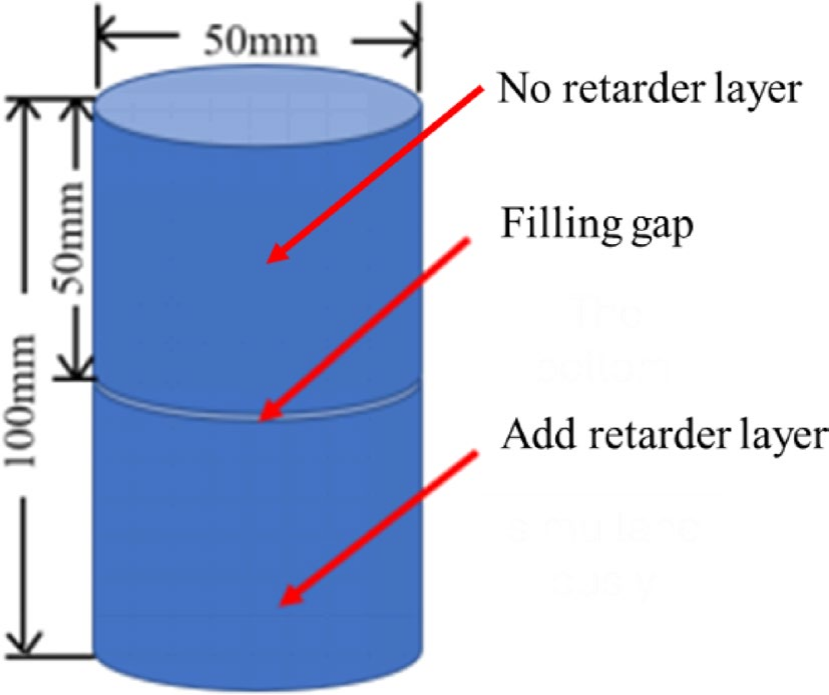
Stratified cemented backfill placement model.

**Figure 5:**
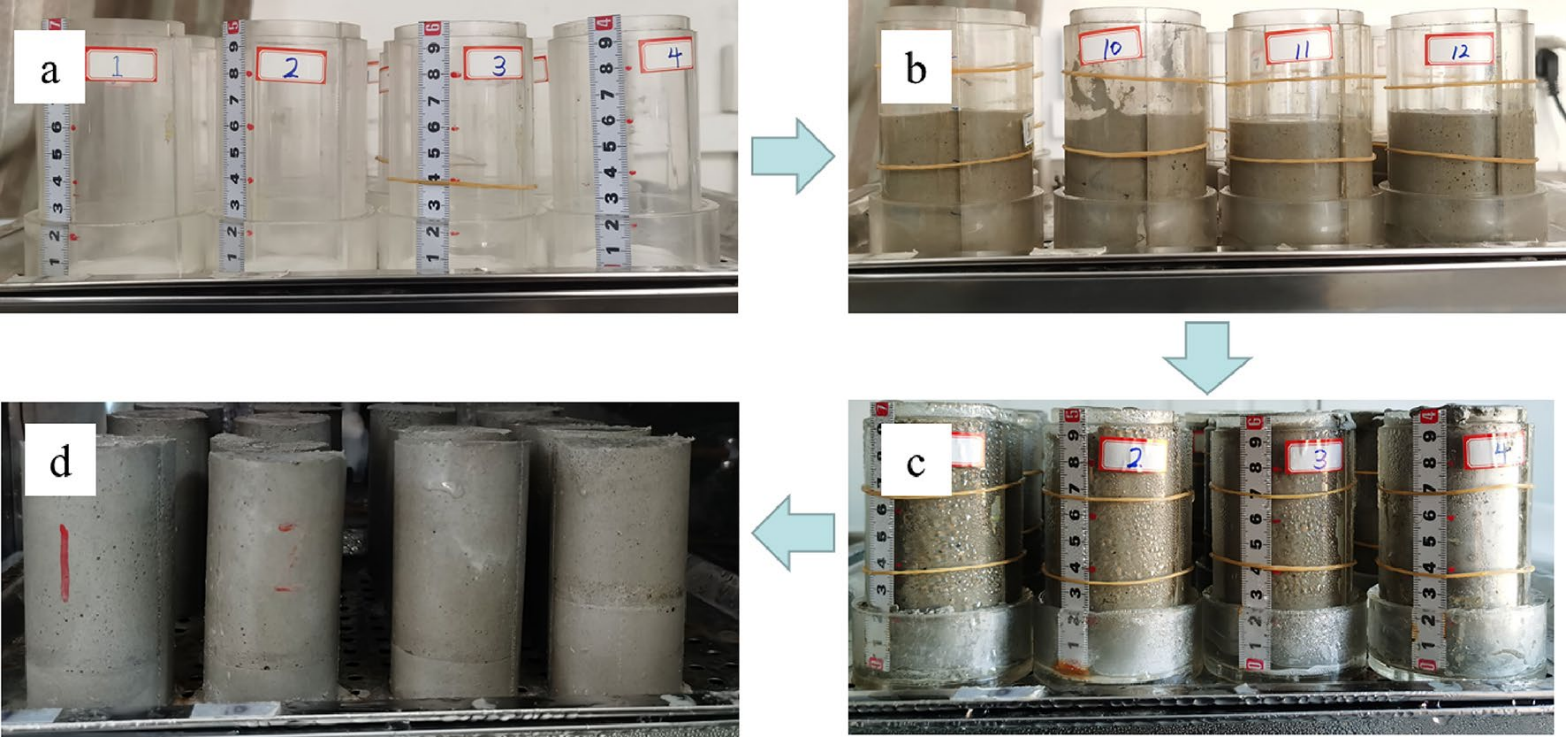
Specimen making process.

Cement filling slurry lime sand ratio according to 1: 6. The mass concentration was set at 75%, and the filling interval time(FIT) was 6 h, 12 h, 18 h and 24 h for layered pouring, as shown in Figs. 4 and 5.

After the pouring was completed, the specimen was cured.The curing temperature of the backfill specimen was 20 ± 2 ℃, and the humidity was above 95%, Then the uniaxial experiment was conducted. TW2000 press system was used in the uniaxial experiment, and the dosage of retarder at different intervals was determined by the setting time of the cemented backfill.

### XRD analysis

The cemented backfill doped with retarder was cured until 28d, washed with anhydrous ethanol to stop cement hydration, ground and dried, and screened through 0.08 mm square holes. The X-ray diffraction patterns of the samples were measured with Merlin, a Zeiss field emission scanning electron microscope, with a scanning range of 10° to 70°.

### SEM analysis

The cemented backfill with retarding agent was cured for 28 days, broken into fast and dried to constant weight. After cooling, the micro-morphology of hydration products was observed by vacuum scanning electron microscope of Rigaku smart lab in Japan.

## Experimental results and analysis

### Study on the setting time of cemented backfill

**Figure 6:**
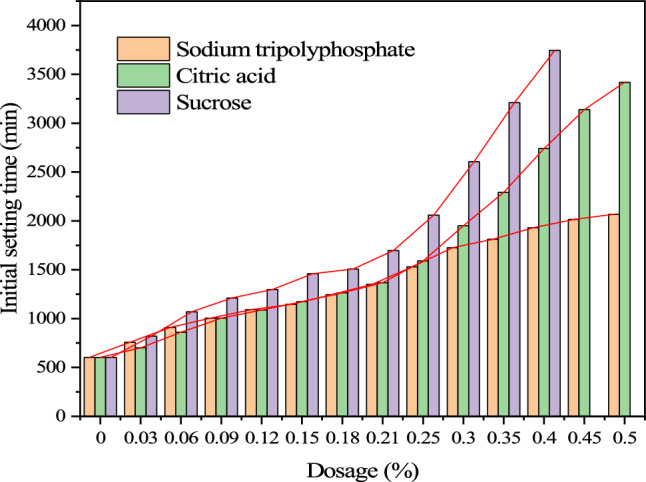
Initial setting time.

**Figure 7:**
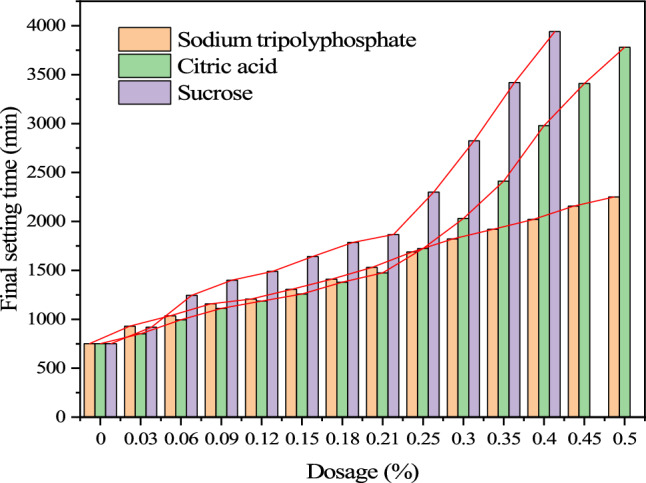
Final setting time.

The influence curve of retarder on the setting time of cemented backfill is shown in Figs. 6 and 7. Retarder has a great influence on the setting time of cemented filling slurry, and the initial setting time and final setting time of cemented filling slurry are extended to a great extent with the increase of different dosage of retarder.

When the content of sodium tripolyphosphate is 0.5% of cement, the initial setting time reaches 34.4 h and the final setting time reaches 37.5 h. When the content of citric acid is 0.50%, the initial setting time reaches 57 h and the final setting time reaches 63 h. When the sucrose content is 0.40%, the initial setting time reaches 62.4 h and the final setting time reaches 65.6 h. Retarding effect Sucrose > Citric acid > Sodium tripolyphosphate. However, when the content of sucrose exceeds 0.40% and the content of sodium tripolyphosphate and citric acid exceeds 0.50%, the cement filling slurry will be difficult to set or the ingredients of the mix will separate from each other.

### Effect of retarder on compressive strength of stratified cemented backfill

According to the determination of setting time, the amount of retarder added at different intervals is shown in Table 2.

**Table 2:** Experimental scheme of strength influence.

FIT	Retarder	Dosage/%
6 h	Sodium tripolyphosphate	0.08
	Citric acid	0.05
	Sucrose	0.04
12 h	Sodium tripolyphosphate	0.2
	Citric acid	0.14
	Sucrose	0.12
18 h	Sodium tripolyphosphate	0.28
	Citric acid	0.22
	Sucrose	0.2
24 h	Sodium tripolyphosphate	0.46
	Citric acid	0.32
	Sucrose	0.25

#### Uniaxial compressive strength analysis

**Figure 8:**
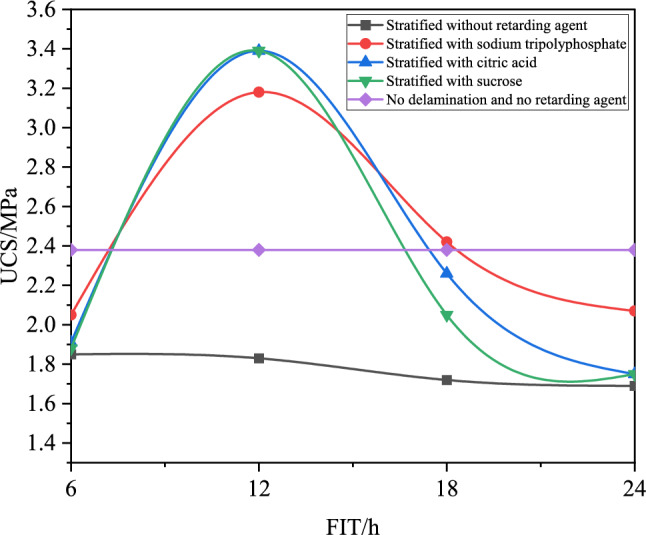
Uniaxial compressive strength of the retarder to stratified consolidated backfill.

The uniaxial compressive strength curve of the stratified consolidated backfill is shown in Fig. 8.The strength of the stratified consolidated backfill can be improved with the appropriate dosage of retarder. Especially when the FIT is 12 h, the strength of retarder-doped layered consolidated backfill is higher than that of unstratified and unretarded backfill, and the effect of citric acid and sucrose is higher than that of sodium tripolyphosphate.

The stratified consolidated backfill containing sodium tripolyphosphate increased by 1.35 MPa and 0.8 MPa, respectively, compared with the stratified consolidated backfill without retarder and the non-stratified consolidated backfill without retarder. The stratified consolidated backfill containing citric acid and sucrose increased by 1.56 MPa and 1.01 MPa, respectively, compared with the stratified consolidated backfill without retarder and the unstratified consolidated backfill without retarder.

#### Failure mode analysis

After uniaxial compression of the specimen of the backfill body, its failure mode is shown in Table 3. When the filling interval is 6 h, the main failure is shear failure, and the cracks run through the delamination, and the delamination cemented backfill with retarder is more complete. When the filling interval is 12 h and 18 h, the main failure is mainly shear-tensile failure, and the main cracks are mostly "Z" through the stratification, and the stratified backfill without adding retarder has more secondary cracks and more serious breakage. When the filling interval is 24 h, the main failure is tensile failure, the main crack bends through the stratification, there are more secondary cracks, and serious shear failure occurs at the stratification. The fragmentation of the stratification backfill without adding retarder is more serious.

**Table 3:** Fragmentation diagram of stratified backfill.

	6 h	12 h	18 h	24 h
No retarding agent				
Sodium tripolyphosphate				
Citric acid				
Sucrose				

## Mechanism analysis

Through the study of the influence of different retarders and different dosage on the physical properties of stratified cemented backfill, the formation mechanism of the strength of stratified cemented backfill was summarized from the quantity of hydration products, the morphology of hydration products and the microstructure of backfill.

### Stratified structural plane analysis

**Figure 9:**
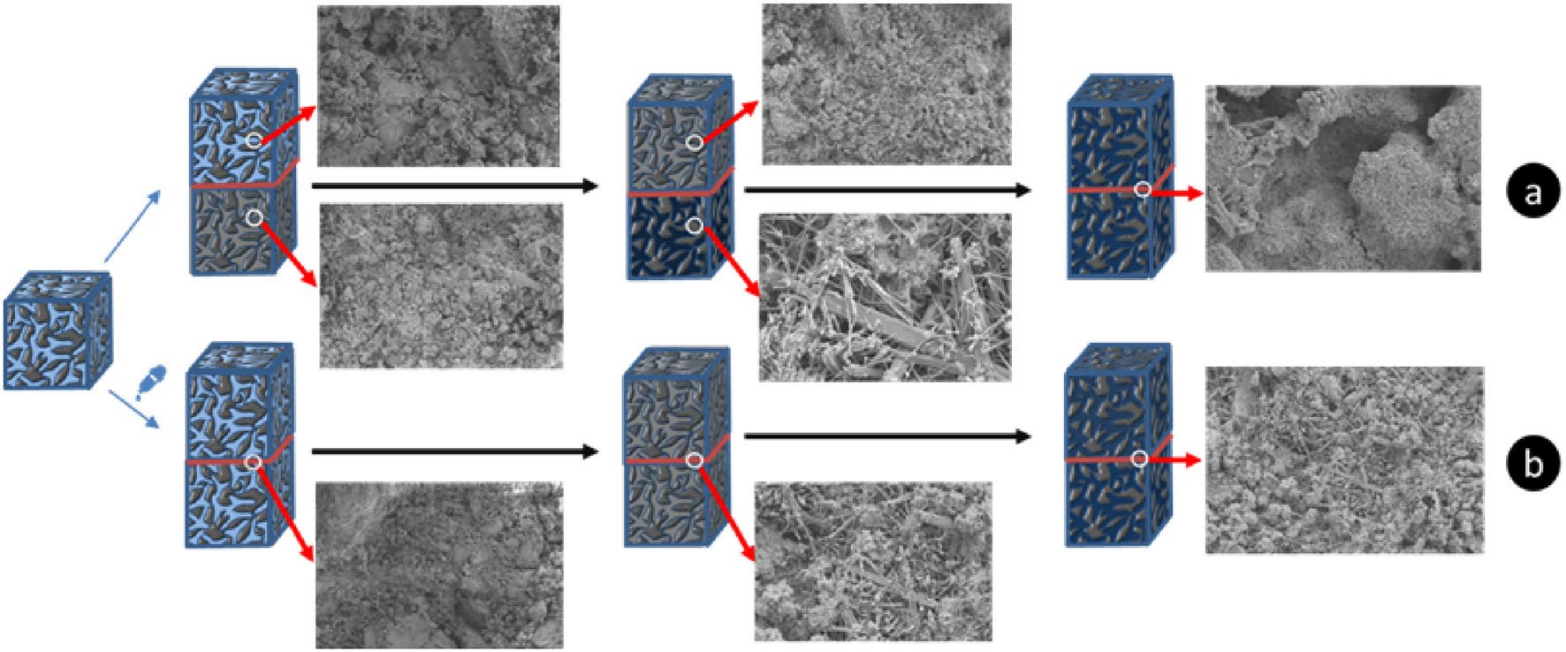
Hydration process diagram of stratified cemented backfill.

The hydration process of the stratified cemented backfill without adding retarding agent is shown in Fig. 9a. The C–S–H structure of the pre-poured filling slurry gradually changes from flock-like shape to needle-like shape, and the hydration activity is greatly weakened or even lost. There are some differences in the structure and cementation properties of C–S–H in the upper and lower backfill during secondary placement at different intervals. As a result, the cementation effect is relatively poor, and the filling gap will appear obvious structural defects.

The hydration process of stratified cemented backfill with retarding agent is shown in Fig. 9b. When retarding agent is added, the lower cemented backfill can remain in the induction period for a long time due to the retarding effect of the retarding agent. C–S–H in the backfill always maintains a floccul-like form that can be fused and cemented with each other. The upper and lower layers of filling slurry can carry out better cementation fusion and enter the acceleration stage at the same time. Finally, the structural defects in the stratification are small, which is conducive to the integrity and stability of the cemented filling body.

### Analysis of morphology change of hydration products

**Figure 10:**
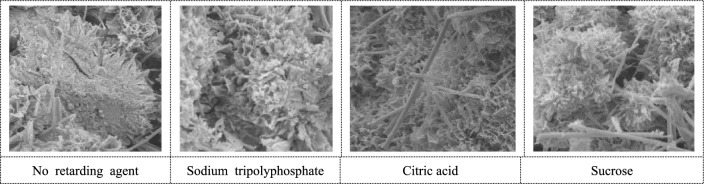
Morphology of C–S–H.

The C–S–H morphology is shown in Fig. 10. In the cemented backfill with retarder, the C–S–H growth is more uniform, the particles are smaller, and the particle density is increased, mainly in the form of fiber and foil.

**Figure 11:**
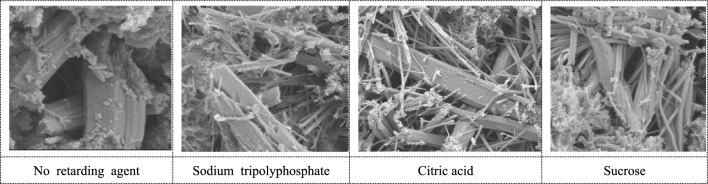
Morphology of CH.

The CH morphology is shown in Fig. 11. The cemented backfill with retarder added has better CH crystallization, and a large number of CH are stacked together. At the same time, needle-rod AFt crystals grow in the pores, which are cross-connected with AFt crystals, without large pores, and are hexagonal plates.

**Figure 12:**
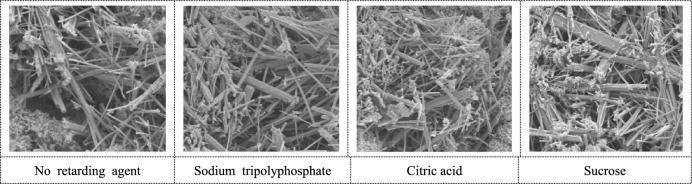
Morphology of Aft.

The morphology of AFt is shown in Fig. 12. In the cemented backfill with retarder, AFt is abundant and interleaved with each other, forming a relatively complete network structure. Cross-linked with C–S–H and CH, AFT forms a relatively dense structure, mainly in the shape of coarse and long needle rods.

**Figure 13:**
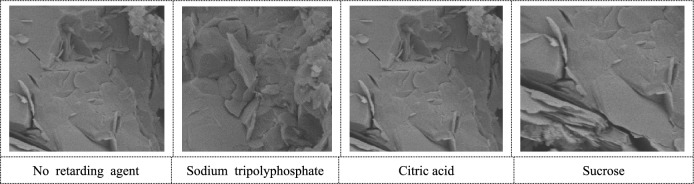
Morphology of Aft.

The morphology of AFm is shown in Fig. 13, which is mainly lamellar and irregular petal-like.

After the addition of different retarders, the micro-morphology, crystallization degree and size of the hydrated products of Portland cement also changed, the hydrated products increased, the hydrated products crossed and connected, the structural compactness increased, and the pores decreased.

### Quantitative analysis of hydration products

**Figure 14:**
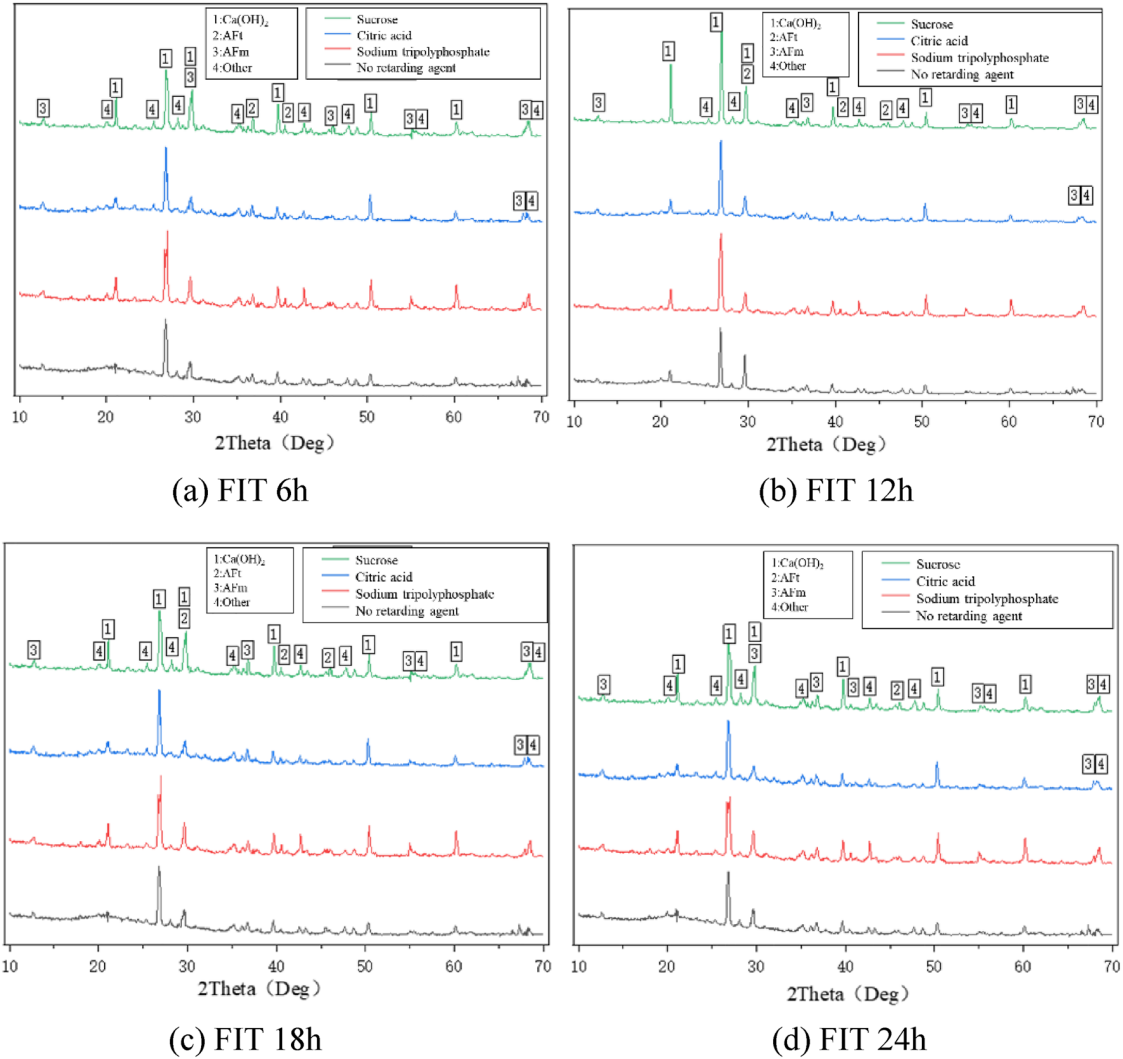
XRD pattern of retarder-doped backfill cement at different FIT. (**a**) FIT 6 h, (**b**) FIT 12 h, (**c**) FIT 18 h, (**d**) FIT 24 h.

As shown in Fig. 14, filling-hardened mortars with different dosage of retarder have similar XRD pattern characteristics. The main hydration product of all samples is Ca(OH)_2_, which has the highest diffraction peak, indicating that its content is large. High diffraction peaks of AFm and AFt can be seen in XRD patterns. In addition, some unreacted tricalcium silicate (C_3_S), dicalcium silicate (C_2_S), tricalcium aluminate (C_3_A), tetralcium ferroaluminate (C_4_AF) and other hydration products (CaCO_3_, etc.) are also found. The Ca(OH)_2_ peak at 12 h filling interval was higher than that at other intervals, indicating that the appropriate dosage of retarder promoted the generation of hydration products, and the dosage of retarder at 12 h filling interval was more appropriate and the strength was the highest. In the appropriate dosage range, three retardants promote the hydration products of cemented backfill: sucrose > citric acid > sodium tripolyphosphate.

## Conclusions

In this paper, Vicat instrument, uniaxial compression test, SEM and XRD are used. The effect of three retarders on the setting time of filling slurry was investigated, and the appropriate dosage of retarder at different intervals was obtained. The influence of retarder on the physical characteristics of stratified filling was studied and its mechanism was explored. The main research contents are as follows:

1. The three retarding agents can delay the setting time of the cemented filling slurry, and the retarding effect is obvious, the retarding effect is sucrose > citric acid > sodium tripolyphosphate. If the cement filling exceeds the appropriate dosage, it will be difficult to condense or the mixture components will be separated from each other.
2. The strength of cemented backfill with appropriate dosage retarder increases first and then decreases with the increase of FIT, and the filling effect is the best when the inter is 12 h. The addition of retarder can improve the integrity of stratified cemented backfill, and the damage of stratified cemented backfill is more complete.
3. Appropriate addition of retarder will increase the number of cement hydration products, change the appearance structure of hydration products, make the structure of hydration products more dense, and make the cementation effect at the stratified cemented backfill better.

## Data Availability

The datasets used and analysed during the current study available from the corresponding author on reasonable request.All data generated or analysed during this study are included in this published article.
